# Late Diagnosis of Partial Androgen Insensitivity Syndrome in a Peruvian Child

**DOI:** 10.7759/cureus.16565

**Published:** 2021-07-22

**Authors:** Marcio José Concepción-Zavaleta, Eilhart Jorge García-Villasante, Francisca Elena Zavaleta-Gutiérrez, José Luis Barrantes Ticlla, Frederick Glenn Massucco Revoredo

**Affiliations:** 1 Endocrinology, Hospital Nacional Guillermo Almenara Irigoyen, Lima, PER; 2 Endocrinology, Hospital Nacional Daniel Alcides Carrión, Lima, PER; 3 Pediatrics and Neonatology, Hospital Belén de Trujillo, Trujillo, PER; 4 Urology, Hospital Nacional Guillermo Almenara Irigoyen, Lima, PER

**Keywords:** androgen-insensitivity syndrome, child, late diagnosis, disorders of sex development, testosterone

## Abstract

Disorders of sexual differentiation are congenital pathologies characterized by atypical development of genetic, gonadal, or phenotypic sex. These are caused by the alteration of any primordial phases of sexual development and may be evident at birth or in the later stage of life. Here, we present the case of a nine-year-old Peruvian school patient who has female gender assigned at birth, has no contributory antecedents and was found to have clitoromegaly and hypospadia on physical examination. In the blood tests, anti-Müllerian hormone and testosterone were found, and 46 XY karyotype and sex-determining region Y (SRY) genes were present. On abdominal ultrasound, testicles were found in the inguinal canals. The human chorionic gonadotropin (HCG) stimulation test was conducted, which allowed us to rule out defects in testosterone biosynthesis and enzyme defects in dihydrotestosterone production; the main suspected diagnosis was partial androgen insensitivity syndrome (PAIS). A multidisciplinary medical meeting was held, accepting the patient’s desire to opt for the male gender, after acceptance by the parents. Thus, the patient underwent bilateral orchidopexy and genitoplasty. He is currently receiving therapy with testosterone, with an adequate response to the treatment and the molecular study confirmed the androgen-receptor gene mutation. In conclusion, we highlight the importance of a timely multidisciplinary diagnosis and management of disorders of sexual differentiation to avoid premature gender assignment and major social and family repercussions that it implies.

## Introduction

Disorders of sexual development constitute a heterogeneous group of congenital conditions that affect urogenital differentiation, causing an atypical chromosomal, gonadal, or anatomical sexual development [1,2]. It can be detected by the presence of ambiguous genitalia in newborns, bilateral inguinal hernias in infants, or atypical secondary sexual characteristics in adolescents [3].

Androgen insensitivity syndrome (AIS) is an X-linked genetic disorder, which is the main disorder of sexual development (DSD) in a patient with a 46 XY karyotype; its prevalence is 1:20,000-1:100,000 births [4]. It is the result of alterations in the androgen receptor gene, leading to a framework of hormonal resistance, which can present clinically in three phenotypes, namely, complete, mild, or partial [5], according to the degree of female phenotype, given the resistance to the biological actions of androgens in male with this karyotype with a normal testicle function and normal concentrations of androgen by age [6].

In this manuscript, we present the case of a patient with a 46 XY karyotype, whose sexual differentiation disorder was diagnosed late.

## Case presentation

A nine-year-old Peruvian patient, who has female gender assigned at birth; is a primary school student and a product of second pregnancy; has no prenatal, natal, postnatal, and contributory family history; has adequate psychomotor development; has no previous medical evaluations; is asymptomatic; and has parents with low educational level, was taken for a control evaluation by a pediatrician. The patient was found to have clitoromegaly on physical examination and was referred to our service for diagnosis and definitive treatment. The parents reported that their son exhibited masculine behaviors and personality traits throughout his life.

On physical examination, the vital signs were observed as follows: blood pressure, 100/70 mmHg; heart rate, 72 bpm; respiratory rate, 18 bpm; and body temperature at axillary level, 36.8 °C. Anthropometry revealed the following: bodyweight, 30 kg (60.57th percentile); height, 128 cm (18.50th percentile); the relationship between height and age, 40th percentile; and body mass index, 18.75 kg/cm^2^ (87.01th percentile). Preferential examination revealed the following: absence of pubic hair, no hyperpigmentation of the external genitalia, glans width of 3 mm, phallus length of 5 mm (clitoral index > 10 mm), presence of urethral meatus in the perineal area, and presence of palpable testicles at the level of the inguinal canal. The rest of the physical examination did not show significant alterations. Among the blood tests initially requested (Table 1), the outstanding findings were the normal value of anti-Müllerian hormone and total testosterone, as well as the 46 XY karyotype, and the presence of the SRY gene. In addition, the LH value indicated that the patient was presumably at a puberal state. X-ray of the left hand reported a bone age of 8.3 years and abdominal ultrasound revealed the presence of testicles in the rudimentary bags at the level of the inguinal canal and the absence of a uterus.

**Table 1:** Blood tests performed during hospitalization R.V: Reference value, ALP: Alcaline phosphatase, AST: Aspartate-aminotransferase, ALT: Alanine- aminotransferase, LH: Luteinizing hormone, FSH: Follicle-stimulating hormone

Blood test	
Complete blood count	Leukocytes: 5,750 cells per cubic millimeter
	Hemoglobine: 13.8 g/dL
	Platelets: 410,000 cells per cubic millimeter
Renal function	Creatinine: 0.39 mg/dL
	Urea: 27 mg/dL
Hepatic profile	Transaminases: AST: 12 IU/L, ALT: 14 IU/L
	ALP: 82 IU/L
	Albumin: 4 g/dL
	Total bilirrubin: 0.8 mg/dL
Hormonal profile	LH: 0.77 mIU/mL (R.V: 0.1-1.1)
	FSH: 2.13 mIU/mL (R.V: 0.3-3.8)
	Basal total testosterone: 20.8 ng/dL (R.V: 3-30 ng/dL)
	Basal total dihidrotestosterone: 9.9 ng/dL (R.V: 3-33 ng/dL)
Anti-Müllerian hormone	55 ng/ml (R.V: 34.3-230.1 ng/mL)
Kariotype	46 XY
Detection of SRY gene by FISH	Present
Blood tests after administration of 1,500 units hCG IM daily for three days	Total testosterone: 47 ng/dL
	Dihidrotestosterone: 10 ng/dL

The diagnostic impression after the initial clinical evaluation suggested an undervirilized 46 XY individual; thus, defect in testosterone biosynthesis, an enzymatic defect in dihydrotestosterone production, or a failure in the testosterone receptor was ruled out. An HCG stimulation test was conducted, which consisted of dosing of testosterone and dihydrotestosterone at baseline and 72 h after the administration of 1,500 units of HCG IM daily for three days, confirming the presence of functioning testicles and ruling out type 2 5-alpha-reductase deficiencies. Consequently, as a result of the studies conducted, the main diagnostic suspicion was partial androgen insensitivity syndrome (PAIS).

The patient was raised as a girl; however, since childhood, the patient presented masculine behaviors. At home, the patient preferred to help with the tasks performed by the father such as heavy farm work and at school, and participated in the activities carried out by the boys. The patient identified himself with the masculine gender and felt uncomfortable with the female appearance. The evaluation by child and adolescent psychiatry did not show alterations in mood or personality disorder. He did not like having long hair or wearing “pink or purple clothes,” as he says.

**Figure 1:**
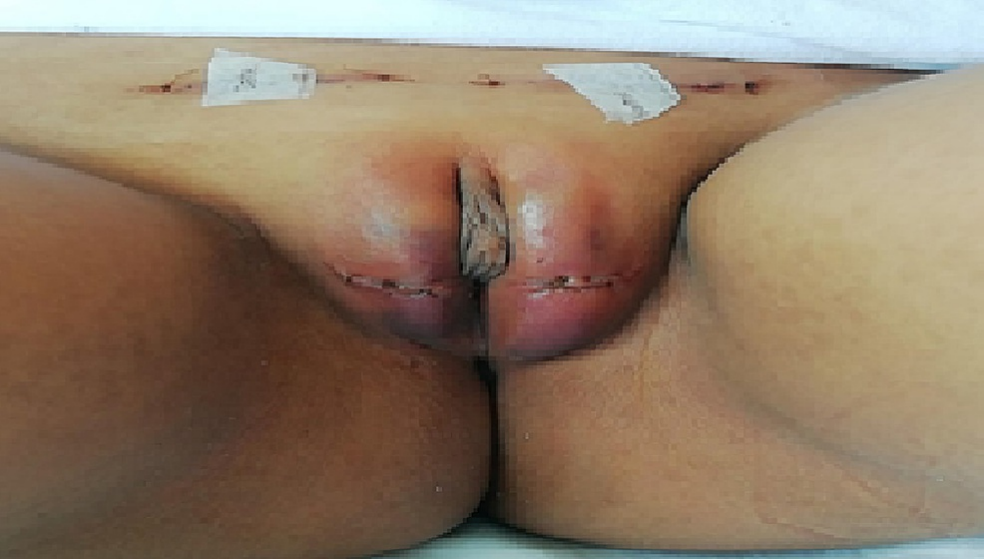
External genitalia in a patient with 46 XY karyotype on the second postoperative day of bilateral orchidopexy.

For this case, a medical meeting was held with psychiatry, endocrinology, pediatrics, and pediatric urology services, in which the patient’s decision to opt for the male sex was accepted, after acceptance by the parents. Therefore, the pediatric urology service scheduled the patient for bilateral orchidopexy (Figure 1), phalloplasty, and scrotoplasty. The patient is currently continuing his outpatient controls on endocrinology, pediatrics, and urology; he receives 125 mg of intramuscular testosterone every two weeks. The molecular study of the androgen receptor confirmed the diagnosis due to an abnormal CAG repetition at the AR gene in the Xq11-12 chromosome. After six months of starting testosterone treatment, the mother has noticed the appearance of pubic hair and penis growth.

## Discussion

AIS is an X-linked genetic disorder, which represents the most common etiology of DSD with a 46 XY karyotype [7]. The alteration is established in the genetic mutation of the androgen receptor, the penetrance degree of which can give three phenotypes, namely, complete, mild, or partial [5].

For all types, the presence of functional testicular tissue with preserved hormonal production is characteristic [7]. Generally, complete AIS presents as an adolescent with a female phenotype, which is evaluated for primary amenorrhea and presents gynecological anatomical alterations such as the absence of uterus or a short vagina, in addition to testes in the inguinal canals. Contrarily, in PAIS, the clinical spectrum will depend on the residual activity of the androgen receptor, i.e., it can vary from a severe lack of virilization, expressed as almost female external genitalia, to an undervirilization that results in varying degrees of external genital masculinization, which typically presents with micropenis, hypospadias, and bifid scrotum with or without cryptorchidism [8].

In the case presented, for the age of the patient, it is still early to see the expected changes typical of puberty. Moreover, despite the fact that the physical examination found subvirilized genitalia, the findings were consistent with PAIS. It has been described that the cases of PAIS that have greater insensitivity to androgens can thus present clitoromegaly in varying degrees and be assigned as female at birth, similar to the case presented here [6].

People with complete AIS lack functional androgen receptors in all tissues, including the hypothalamus and the pituitary gland. Consequently, these individuals are resistant to negative androgen feedback in the pituitary. A supranormal luteinizing hormone (LH) would reflect the need for a specific androgenic action in the negative feedback of gonadotropin secretion. In PAIS, as there is a variable response of the androgen receptors, the negative feedback at the central level is not uniform. Compared with PAIS patients, complete AIS patients exhibited higher basal FSH, peak FSH, and peak LH hormone levels but lower AMH expression [9]. Thus, gonadotropins may be in normal ranges, as in the case of our patient [7]. Infants with PAIS have a more sensitive pattern of testosterone at baseline and after HCG stimulation. Therefore, this test is a key to the exclusion of other causes, which are part of a differential diagnosis such as defects in androgen production (e.g., gonadal dysgenesis, LH mutations, or biosynthetic enzyme deficiencies) or genetic causes [10].

The normal ratio of total testosterone/dihydrotestosterone post-stimulation with HCG is less than 10; higher values suggest type 2 5-alpha-reductase deficiency [11]. In the patient, the values obtained for these hormones after conducting the test were in a ratio of 4.7, and the possibility of this enzyme deficiency was ruled out. Likewise, the normal range of anti-Müllerian hormone, the normal value of dihydrotestosterone, and the normal response of testosterone to the HCG stimulation test ruled out the possibility of testicular dysgenesis. In this way, all these findings place us in the context of PAIS [12].

However, it has been shown that the repercussions in the child, and later in the adult, are not only limited to a poor or null reproductive capacity; the quality of life of these patients is also significantly affected. In this regard, it has been described that the most compromised areas are self-esteem, physical well-being, and school performance [12]. In the mental sphere, people with DSD were found to suffer from anxiety (19.5%), depression (7.1%), attention deficit hyperactivity disorder (4.1%), and autism (9.1%) [13]. Remarkably, a study reported that the most severe psychiatric symptoms were found in patients with DSD who had Y chromosomes, among which is the PAIS [14]. In our case report, the psychiatric evaluation found distress related to his physical appearance and the concept of himself, confirming gender dysphoria [15]. As previously described, the patient has a male gender identity, as the proof of this is that the patient independently chose a masculine name to be called [16]. The genetic sex of the patient was accepted by himself and his parents gave consent to the surgical procedures performed on him.

Due to the possible repercussions, the patient´s gender must be assigned as early as possible to establish their sexual identity, and given this, the treatment is multidisciplinary and must include specialists in endocrinology, surgery, urology, and mental health and also the participation of the parents [17].

In the case of PAIS, surgical and hormonal treatment is aimed at the agreed gender assignment. The current guidelines recommend that if the patient was raised as female, gonadectomy should be performed, and estrogen therapy should be provided [18]. Conversely, if the patient was raised as a male, the testicles should be preserved, and orchidopexy should be performed, in addition to hormonal therapy with androgen [7]. The role of long-term testosterone therapy in individuals with PAIS who are raised as males remains unclear. Response to androgen treatment may be substantial in individuals with certain missense variants in the DNA-binding domain of the androgen receptor [19]. A recent study confirmed the difficulty of accurately predicting the efficacy of androgen treatment [20]. Our patient currently is responding to testosterone treatment, as evidenced by the development of secondary sexual characteristics.

## Conclusions

In conclusion, the present case suggests the importance of a timely multidisciplinary diagnosis and management of disorders of sexual differentiation to avoid premature gender assignment and major social and family repercussions that it implies.

Likewise, we highlight the relevance that in patients with DSD, in whom the suspicion of PAIS is high, it is recommended to study the androgen receptor mutation, to confirm the diagnosis, due to the repercussions in the treatment to which it is associated.
